# Commencements

**Published:** 1895-04

**Authors:** 


### COMMENCEMENTS.

The thirty-seventh annual commencement of the Atlanta Medical College was held at the Grand Opera House April 2. The address was delivered by Professor H. C. White, of Athens, and was elegant in every way. In the annual report of the proctor, Dr. W. S. Kendrick, it was shown that the College had grown and prospered during the last year in spite of the hard times. One year ago, in accordance with the demand for better medical education the three-years' course, of six months each, had been adopted. The session just closed had been the most successful in the history of the College. In all departments—medical, dental, and pharmaceutical—there had been three hundred and eighty-three students in attendance. The graduating class numbered one hundred and thirty-five. Upon these the degree of Doctor of Medicine was conferred by Hon. N. J. Hammond, President of the Board of Trustees. Dr. J. C. King was the valedictorian of the class. Dr. J. W. Torbett, of Texas, was awarded the first honor, having made the highest average in the history of the College. Dr. E. C. Cartledge and Dr. H. McCulloh received second honor, and Dr. W. J. Shaw and Dr. C. L. Youmans third honor. After delivery of the prizes by Mr. Hammond the very pleasant exercises were closed.

The Southern Medical College closed its sixteenth session on the evening of April 3d at the Grand Opera House. After prayer by Dr. Holderby, Dr. William P. Nicolson, Dean of the College, made his report, showing that the usual number of students, about one hundred, had been in attendance during the session. Two years ago the faculty had voluntarily adopted the three-years' course, together with the demand that the student should have a good English education. On account of these requirements the College presented the smallest graduating class that has gone out from the institution in fifteen years, the small proportion of graduates to the body of the class being a just cause for pride. The graduating class numbered sixteen. The diplomas were presented by Dr. T. S. Powell, President of the Board of Trustees. In the absence of Hon. W. C. Glenn, who was to have delivered the annual address, this duty was performed by Professor J. McFadden Gaston. The first honor man of the class was Dr. H. E. Russell, of South Carolina; the second honor, Dr. E. P. Rumph, of Texas; the third, Dr. T. C. Longino, of Georgia. The valedictory was delivered by Dr. Charlton Shaw, of Canada.

